# Study Protocol for a Randomized Controlled Trial Assessing the Effectiveness of Personalized Computerized Cognitive Training for Individuals With Insomnia

**DOI:** 10.3389/fnbeh.2022.779990

**Published:** 2022-02-28

**Authors:** Jose Luis Tapia, Francisco Javier Puertas, Jon Andoni Duñabeitia

**Affiliations:** ^1^Centro de Investigación Nebrija en Cognición (CINC), Universidad Antonio de Nebrija, Madrid, Spain; ^2^Unidad Sueño, Hospital Universitario de la Ribera-FISABIO, Valencia, Spain; ^3^Facultad de Medicina, Universidad Católica de Valencia, Valencia, Spain; ^4^AcqVA Aurora Center, The Arctic University of Norway, Tromsø, Norway

**Keywords:** insomnia, cognitive stimulation, cognitive intervention, sleep disorder, sleep quality, cognitive performance, quality of life

## Abstract

Insomnia is a frequent and heightened pathology in the general population of developed countries, and its condition generally leads to health discomfort and performance drop in daily and work-related tasks. As current pharmacological treatments for insomnia do not always seem sufficient to mitigate impairment, contemporary cognitive approaches might shed light on developing complementary therapies for this population. We propose a cognitive stimulation intervention program based on the importance of cognitive abilities as precipitating and maintenance variables of sleep disturbances. A full phase I-II-III clinical trial is proposed in which the first two studies will serve to assess the safety of the intervention and to identify the maximum tolerated time of the computerized cognitive training (phase I) and the minimum effective number of training sessions (phase II) in the absence of adverse events or side effects. Next, a phase-III double-blind randomized controlled trial design will be set. Sixty individuals with insomnia aged 25 to 55 years will enroll in a home-based personalized computerized cognitive stimulation program for a total time of 8 weeks, training 5 days per week. Sixty insomnia patients matched in a variety of factors will constitute the active control group, where the orthogonal activities will not be cognitively demanding. Sleep, cognitive, emotional, and quality of life variables will be measured before and immediately after training. A linear mixed model and hierarchical regression analysis will be used to investigate intervention effects. The results derived from this study will be precious for future research and treatment in cognitive performance and clinical pathologies.

**Clinical Trial Registration:** [https://clinicaltrials.gov/], identifier [NCT05050292].

## Introduction

Insomnia is a clinical condition characterized by a difficulty falling or staying asleep, or going back to sleep after morning awakenings, even when optimal environmental conditions are present. In addition, this situation must occur without apparent psychiatric or somatic causes (Thorpy, 2017). In recent decades, the prevalence of this disorder has increased in industrialized countries, estimating that around 12% of the general population suffers from insomnia (Grewal and Doghramji, 2017), reaching 60% when it occurs comorbidly (Morin et al., 2006). Insomnia or poor-quality sleep derived from insomnia negatively impacts cognitive performance, mainly affecting executive functions, attention, memory and concentration (Ilioudi et al., 2011). Although there is some controversy among the cognitive domains affected by insomnia, the main cognitive skills where there is consistent poor performance are those assessed by tasks that measure attentional control, working memory, and problem solving, which are part of the cognitive processes involved in executive functions (see Fortier-Brochu et al., 2012; Miyata et al., 2013; Ballesio et al., 2019; Wardle-Pinkston et al., 2019). Likewise, insomnia has also been associated with poor academic/work performance (Bolge et al., 2009), a greater propensity to suffer occupational accidents (Kessler et al., 2012), and a greater vulnerability to other medical or psychiatric disorders (Chien et al., 2010; Khurshid, 2018), all these factors clearly affecting the quality of life of the patients.

### Explanatory Models

Since the 1980s, insomnia research has pinpointed negative intrusive thoughts and worries about sleep as the leading cause of difficulty initiating sleep (Borkovec, 1979, 1982). Subsequently, several studies have indicated that individuals with insomnia are prone to anxiety, worry, obsession, and hypervigilance during the day (Kales and Kales, 1987; Edinger et al., 1988; Morin, 1993). These discoveries gave way to cognitive models of insomnia, such as the one presented by Harvey (2002). According to this model, the presence of rumination and cognitive concern about not being able to fall asleep or not getting enough sleep to function the next day leads to a state of anxiety with the subsequent activation of the sympathetic nervous system. Consequently, attentional activity will focus on looking for potentially harmful stimuli, increasing brain activity, and feeding back concerns and worry about falling asleep. This fact of paying attention to internal and external stimuli and sensations has been contrasted using neuroimaging and neuronal activity recording techniques, supporting the central hypothesis of brain hyperactivation that underlies this model (Bastien et al., 2021).

### Neurobiological Bases of Insomnia

Over the years, predisposing, precipitating, and maintaining factors of insomnia have been defined (Bastien et al., 2004b). Researchers and clinicians alike have relied on neurocognitive (Perlis et al., 1997), cognitive (Harvey, 2002), and psychobiological inhibition models (Espie, 2002) to try to understand insomnia better. Interestingly, one approach that is gaining increasing attention to discover specific details of the pathophysiology of this disorder is neurobiology.

Throughout the past decades, the presence of cortical and physiological excitation and difficulties in inhibitory processes in insomnia have been highlighted in several studies carried out using different neuroscientific techniques. At a morphological level, research on structural magnetic resonance imaging (MRI) shows a reduction in the volume of gray matter in the medial frontal lobes, the hippocampus, the parietal cortex, and the anterior cingulate cortex in patients with insomnia (see Riemann et al., 2015), implying plausible memory impairment and frontal lobe dysfunction. According to Killgore et al. (2013), the observed increased functional connectivity between motor and sensory areas may explain the increment of unwanted and uncontrolled sustained and sensory awareness and processing. In addition to the structural alteration, functional magnetic resonance imaging (fMRI) studies have revealed greater activation in the hippocampus and the medial frontal gyrus (Leerssen et al., 2018) and reduced blood flow of the default neural network (DMN), associated with the modulation of conscious processes (Santarnecchi et al., 2018). These findings are consistent with the hyperarousal theory of insomnia (see Riemann et al., 2010, for review), supporting the increased mental worry and rumination characterized in the sleep onset and sleep maintenance of individuals with insomnia.

Research in electroencephalography (EEG) is congruent with the dysfunctional worries and attentional focus that these individuals experience (Harvey and Tang, 2012). Different studies have reported higher beta wave activity (associated with alert states) and lower delta activity (related to drowsiness) at the onset of sleep (e.g., Freedman, 1986; Merica and Gaillard, 1992), and higher alpha, beta, and sigma activity together with lower delta activity during REM sleep (Krystal et al., 2002). In line with the fMRI studies on the DMN, Corsi-Cabrera et al. (2012) found impaired frontal deactivation of cortical regions involving cognitive functions related to attentional control, stimulus inhibition, and self-awareness. The state of wakefulness present on sleep onset has also been explored through positron emission tomography (PET), finding an increased alertness brain activation (Spiegelhalder et al., 2017). Furthermore, studies with event-related brain potentials (ERPs) show that patients with insomnia present greater amplitude of the P1 waveform (an ERP component associated with the cognitive cost of care) and the N1 component (related to the detection of stimuli) upon waking up (Loewy and Bootzin, 1998; Loewy et al., 1999; Bastien et al., 2008), and greater amplitude of the P300 component (an ERP component that reflects the update of active working memory) at the beginning of sleep (Hull, 1993). At the same time, a decrease in P2 waves has been observed upon awakening (Loewy and Bootzin, 1998; Loewy et al., 1999), and a lower N350 at the beginning of sleep (Bastien et al., 2008), both components being associated with inhibition of the processing of irrelevant potentially disturbing sleep stimuli. ERPs results are consistent with EEG and PET studies, observing cortical hyperactivation and hypervigilance and low cognitive inhibition performance. These cognitive deficits in both activation and inhibition functions interfere with the mechanisms to initiate and maintain sleep, thus hindering people’s chances of falling asleep, which translates into insomnia.

### Insomnia Treatment

The first-line treatment for insomnia, stated by different clinical practice guidelines such as the American College of Physicians (ACP) and the European guide for the diagnosis and treatment of insomnia, is Cognitive Behavioral Therapy (CBT, or CBT-I for the insomnia variant) (Riemann et al., 2017). However, the most used treatment for people with insomnia remains medication (i.e., benzodiazepine, benzodiazepine receptor agonist, and non-benzodiazepine hypnotics), probably because of the lack of knowledge of alternatives by primary-care physicians and the generalized search for immediacy typically achieved with pharmacotherapy (see Koffel et al., 2018). Given the chronicity of insomnia and the well-known high addiction risk and long-term side effects of medication (Benca, 2005; Buscemi et al., 2007), non-pharmacological interventions should prevail in these patients.

In a general sense, CBT-I therapy seeks to readjust maladaptive or misleading cognitive beliefs related to sleep through the following main components: (a) relaxation, focused on teaching patients to generate a relaxation response (i.e., slow breathing, reduced heart rate) contrary to the stress response that increases arousal levels; (b) sleep hygiene and stimulus control therapy, where psychoeducation is used to instill healthy behaviors related to sleep, such as avoiding large dinners, not using digital screens at night, or limiting excessive light or noise; and (c) sleep restriction, aimed to match sleeping periods with the adequate sleeping time by forbidding patients to sleep during daytime hence accumulated sleeplessness will force nighttime sleeping.

Despite the extensive literature that corroborates the effectiveness of CBT-I (Koffel et al., 2015; Trauer et al., 2015; Sato et al., 2019), this therapy presents significant setbacks, including the stigma or reluctance in receiving psychological treatments, the in-person appointments, and the need to be performed by a qualified therapist. Given the shortage of professionals trained in CBT-I, availability is limited, and in most cases, it requires the patient to travel long distances. To avoid these primary accessibility impediment, the standard face-to-face CBT-I intervention was adapted to be administered by telephone, while maintaining adequate effectiveness (Bastien et al., 2004a). Still, the need for a therapist continued limiting access because of the waiting lists, so in recent years CBT-I has been extended and computerized to be administered digitally and *via* online. In this sense, CBT-I has been developed as (1) a support tool for conventional therapy, where the patient has access to teaching material and exercises to be completed at home, (2) an autonomous intervention guide with sporadic support from trained healthcare personnel, and (3) an individualized, unguided, and fully automated intervention—namely self-help intervention. However, even though these digital applications have been shown to be effective in clinical trials (see Seyffert et al., 2016; Luik et al., 2017; for review), the complexity of some tasks in the absence of a therapist and the required self-commitment and self-discipline by the patient compromises treatment adherence, reporting in some clinical studies drop rates over 40% (Vincent and Hameed, 2003; Ong et al., 2008). Together with this, it is also worth noting that the normative rules and rigidity of the sleep restriction component of the CBT-I seems to be counterproductive in some patients, diminishing their motivation and leading to diurnal fatigue and sleepiness (Kyle et al., 2014).

Over the years, there have been other non-pharmacological approaches showing promising results. A recent review by Chan et al. (2021) on non-pharmacological interventions in insomnia mentions mindfulness or Tai Chi as possible alternatives or complementary practices to treat sleep disturbances. Nevertheless, the objective of these practices is comparable to the relaxation/meditation component of CBT-I, seeking better knowledge and perception of internal and external stimuli, a greater focus on attentional processes, and, therefore, an improvement in the inhibition of other stimuli considered irrelevant or potentially harmful. Currently, both mindfulness and Thai Chi interventions have functional digital support applications. However, as in the case of CBT-I, adherence and therapy commitment are compromised due to complexity, abstraction, patient reluctance, and low level of motivation (Li et al., 2004; Ong et al., 2014; Black et al., 2015).

As the key component of insomnia seems to be hyperarousal and executive function deficit, interventions that enhance cognitive functioning (primarily inhibition mechanisms and attentional processes) might effectively reduce brain hyperactivation and distorted attentional stimulus focus. Even though the CBT-I might entail cognitive skills training, its main purpose is to restructure distorted cognitive beliefs and give the patient comprehensive safety behaviors, leaving aside the cognitive functioning alterations (i.e., attention, memory, and executive functions) present in patients with insomnia. In this sense, in recent years, computerized cognitive training (CCT) programs that reinforce the neural connections of the cognitive mechanisms underlying attentional control and inhibitory processes have been proposed as a successful method to directly focus on the dysfunctional cognitive mechanisms by stimulating the critical memory and attention cognitive domains, resulting in better ease of falling asleep.

The CCT approach is based on the concept of cognitive stimulation, aimed to strengthen neural connections through cognitive training by completing and repeating digital activities or exercises designed to improve cognitive skills with a mental workout (see Tapia and Duñabeitia, 2021). Numerous studies have demonstrated that cognitive training activities have remarkable positive results both in healthy population (Shatil, 2013; Bonnechère et al., 2020) and in different pathologies (e.g., Shatil et al., 2010; Preiss et al., 2013; Reijnders et al., 2013; Lawlor-Savage and Goghari, 2014; Spreij et al., 2014; Zhang et al., 2019) when assessing cognitive performance, even though the evidence showing far transfer effects is limited (e.g., Rebok et al., 2014; D’Antonio et al., 2019). However, an area of study in which CCT has been shown to be an effective method is that of sleep-related pathologies, evidencing improvements not only in the cognitive functioning but also in self-reported sleep quality when administered together with physical activity (Pa et al., 2014), and when compared to psychoeducation (Diamond et al., 2015; Almondes et al., 2017). To date, most studies that refer to cognitive training in the context of insomnia use the strategies and components of CBT-I but do not follow a proper CCT approach. For instance, Keramtinejad et al. (2019) proposed an 8-week cognitive training intervention to improve cognitive functioning and sleep quality of older adults with chronic insomnia. Although the intervention program included training of cognitive functions such as memory, attention, or executive functions, the proposed activities’ content was delivered in the form of group discussion or relied on cognitive therapy approaches. Therefore, due to the little coverage of pure cognitive rehabilitation in CBT-I interventions, it is worth considering CCT as an aid for patients with insomnia.

In this line, Haimov and Shatil (2013) provided the first piece of evidence on the effectiveness of CCT in insomnia. The intervention of their study consisted of a home-based personalized and computerized cognitive training program using a commercial software and was carried out with seniors who suffered from insomnia. Participants were randomly assigned to either an experimental group that received personalized training, or to an active control group that was instructed to perform different tasks in typing and painting commercial software. To our knowledge, the study by Haimov and Shatil is the only existing one that uses CCT without applying cognitive therapy in individuals with insomnia.

## The Current Proposal

Given the disadvantages of pharmacological treatment for insomnia in the long term and the high costs of CBT-I, and considering the usefulness of computerized cognitive training (CCT) as an aid, the development of new lines of intervention in this perspective is deemed crucial. Based on the existing literature on the precipitating and maintenance factors of insomnia, we propose a research agenda with the proposal of a clinical trial aimed at demonstrating the safety and efficacy of an intervention based on a personalized CCT focused on the improvement of the cognitive components related to the processes of inhibition, attention, perception, and processing of stimuli, in order to partially compensate the problems related with cognitive states of concern and alert typically present in patients with insomnia disorder. To this end, a full phase I-II-III study protocol is presented. While the core of the intervention will be the phase-III randomized controlled trial, a safety and dose-limiting analysis protocol has also been designed as part of the phase-I/II trials to provide informed decisions. A phase-I trial will be conducted in order to assess the safety of the CCT and to determine the maximum tolerated training time per session (equivalent to the maximum tolerated dose of a medicine). Next, a phase-II trial will also be run to validate the safety of the CCT while determining the minimum necessary training sessions to observe significant effects (namely, to test the efficacy of the CCT). Finally, the critical phase-III trial will be carried out through an online platform of personalized CCT programs (CogniFit Inc., San Francisco, CA, United States), which will be administered to a group of patients with insomnia, while a different group of participants of similar characteristics will complete a control intervention lacking high cognitive demands and based on the completion of low-level computerized artistic tasks.

This intervention could be of great help for patients with mild sleep problems or, if combined with CBT-I, for more severe cases. In addition, the proposed methodology allows access to the intervention to people with limited resources and those who reside in rural or isolated areas, given that it can be carried out from anywhere with an Internet connection and a smartphone, tablet, or computer. The main difference between our proposal and other treatments (and hence the reason for its value as a complementary approach) is that the intervention does not focus directly on the observable symptoms of insomnia but rather on the core underlying cognitive mechanisms that modulate them. Thus, the proposed CCT-based intervention aims to enhance executive functions and attentional processes, reduce monitoring of sleep-related threat signals, and improve inhibition processes of disruptive stimuli.

## Objectives and Hypotheses

Given the findings that show that the excessive cognitive activity present in patients with insomnia is due to a deficit in the functioning of various cognitive functions related to attention and executive functions, we hypothesize that a systematized training in such functions will act as a catalyst on cortical hyperactivity, limiting rumination, intrusive thoughts and disturbing stimuli, thus improving the quality of sleep. We postulate that insomnia patients will also benefit from a general cognitive performance enhancement when CCT mainly targets cognitive functions.

Therefore, the present study has three main objectives: (1) establish the acceptable sessions’ range to determine the maximum training time before fatigue or other adverse conditions appear; (2) determine the therapeutic efficacy of the intervention, establishing the range of needed training sessions, the tolerability to exercises, and the safety of the activity; and (3) verify that CCT can be a plausible non-pharmacological intervention to improve sleep quality in insomnia individuals.

## Methods and Analyses

### Design

#### Phase I

A standard 3 + 3 rule based up-and-down design without dose de-escalation will be followed (see Lin and Shih, 2001). This will be an open label prospective phase-I dose-escalation trial. Candidate participants will be recruited from the Sleep Unit of the Hospital Universitario de la Ribera (Spain). All participants will be individually interviewed in order to verify that they meet the inclusion criteria and to check their availability for the study. All participants will electronically sign an informed consent form prior to the beginning of the scientific actions. Together with the consent form, they will receive information about the approval of the protocol by the Ethics Committee in which the alignment with the criteria set by the declaration of Helsinki will be explicitly indicated. At all moments of the procedure, patients will be accompanied by trained medical doctors and psychologists from the research group.

All participants will complete a first session in which they will respond to a general sociodemographic and medical questionnaire to ascertain that they meet the inclusion criteria. Together with this, they will also complete a general cognitive and psychological evaluation lasting for 60 min using either a laptop, PC or tablet. The instruments used for this assessment are described below (see *Instruments and Outcome Measures* section).

The first cohort of participants (*N* = 3) will then start the phase I testing protocol. All participants will complete CogniFit Personalized Online Training, consisting of individual sessions in which three brain training games with a duration of around 5 min each need to be completed. The CCT will train six fundamental cognitive skills associated with the domains of memory, attention, perception and coordination: naming, working memory, non-verbal memory, visual perception, response time, divided attention and inhibition. The three participants from the first cohort will individually complete a 15-min training session under the supervision of a researcher from the team and a clinical psychologist. After finishing the session, they will complete a fatigue and safety evaluation questionnaire aimed at obtaining information about potential side effects and adverse events associated with the clinical process (see *Instruments and Outcome Measures* section). In case no side effects or adverse events are reported, a dose escalation will be implemented, and participants will then continue with a new training session of similar characteristics, structure and duration. The fatigue and safety questionnaire would be repeated right at the end of this new session, and the protocol would cyclically continue until the supervising researchers decide not to continue with an additional session because of having reached the dose-limiting toxicity level. If one participant from the cohort reports extreme fatigue levels (scores of 9 or 10 on the scale described below) or side effects or adverse events (see the questionnaire below) with a certain dose level (level i), a new cohort of three participants would start the protocol with the same dose level. If no other participant from the 3 + 3 cohort reports extreme fatigue, side effects or adverse events, the procedure would restart for a new cohort of three participants at the next dose level (level i + 1). However, if more than one participant from the 3 + 3 pool reports any of the outcomes, the maximum tolerated dose of the CCT would be set at level i-1. If two or more participants from the first cohort report extreme fatigue levels, or side effects, or adverse events with a certain dose level (level i), the maximum tolerated dose of the CCT would be set at level i-1.

#### Phase II

This trial will include a different group of 20 patients from the same hospital who meet the inclusion criteria described above, who will also sign the corresponding consent forms prior to initiation of collaboration. After having set the maximum tolerated training time per session in the phase I trial, a 15-day phase-II trial will be designed to ascertain the effectiveness of the CCT in the absence of adverse events or side effects.

To this end, 20 patients from the Sleep Unit of the Hospital Universitario de la Ribera (Spain) will be recruited and they will first complete an initial session (Day 1) that will serve to collect baseline data about their sleep problems and their cognitive skills, as well as about their sociodemographic information. As in the phase I trial, they will complete the general cognitive and psychological evaluation lasting for around 60 min (see Instruments and Outcome Measures section).

The day after the initial evaluation (Day 2), participants will be asked to visit the Sleep Unit again to complete the first training session with the CCT. The duration of the training session will be determined by the results of the phase I trial. Participants will complete this training session using either a tablet, a PC or a laptop, accompanied by the research and clinical team. Right at the end of the session, and in order to assess the safety of the intervention, participants will also complete the same safety questionnaire as in the phase I trial. In case any participant at any moment would report potential adverse events in any of the safety questions, the clinical psychologist would immediately interview them in order to classify the side effect or adverse event according to its seriousness following the criteria and elements adapted from the Patient-Reported Adverse Drug Event Questionnaire (de Vries et al., 2013).

The protocol would then continue the next day (Day 3) with a full assessment identical to that of the first session. The day after (Day 4) would consist of the repetition of the training session of Day 2. This way, and alternating assessment and training sessions, participants will complete a 15-day cycle that will start and end with a full assessment (Day 1 and Day 15), and that will include eight different evaluation or test points (Day 1, Day 3, Day 5, Day 7, Day 9, Day 11, Day 13, and Day 15) and seven training sessions (Day 2, Day 4, Day 6, Day 8, Day 10, Day 12, and Day 14).

The results of the effectiveness of the CCT will be evaluated using linear mixed models for repeated measures, analyzing differences in the primary and secondary outcomes described below. This will provide a first approach to and statistical validation of the usefulness of the CCT to alleviate the cognitive, psychological or medical symptoms of insomnia in the selected sample by determining the minimum number of training sessions needed to observe a significant change. Besides, quantitative and qualitative analysis of the results of the safety questionnaires will help us determine the safety of the intervention program.

#### Phase III

The final stage of the protocol will consist of a double-blind randomized controlled phase-III trial in which a large group of patients with insomnia recruited from the Sleep Unit of the Hospital Universitario de la Ribera, as well as from collaborating medical institutions will voluntarily enroll in an 8-weeks personalized and supervised cognitive training intervention. The medical team will inform those patients who meet the inclusion criteria of the possibility of voluntarily participating in the experiment. Those interested will be put in contact with the researchers, who will interview them individually. In this first contact, all the information regarding the study will be provided. Patients who agree to participate will complete the necessary legal documentation and informed consent and be included in the study.

A complete evaluation will be carried out at the beginning (pretest) and at the end of the intervention (post-test) using the materials and instruments also used in the phase-II study. The pretest will collect baseline data about sleep problems and cognitive skills, together with sociodemographic information. Standardized batteries and tests will be used to measure the general neurocognitive state and the emotional state, together with specific tests and measurements of the pattern and quality of sleep, and tests that measure health-related quality of life. All variables will be measured objectively (i.e., direct test scores) and subjectively (i.e., what the patient or her close associates express). The post-test will collect data on sleep problems and cognitive skills, but no additional sociodemographic data will be needed (see *Instruments and Outcome Measures* section for a description of the tools). Having already established the safety of the protocol in the phase-I and phase-II studies, no information will be collected in this regard in the phase-III trial.

Next, the CCT intervention will be carried out. This will be done through the CogniFit online platform so that each participant will access it from home using a personal computer or smart device. The training will last for a total of 8 weeks, with five training days per week. This way, a total of 40 training sessions are expected. The duration of the sessions will be determined by the maximum tolerated training time resulting from the phase-I trial. Since this process will be autonomously managed by each participant, they will be contacted periodically (a minimum of two phone calls per week) to ensure adherence and check that they have not had any incidence. The platform will record participants’ performance, providing researchers with real-time feedback on the performance and adherence of each participant.

Participants will be randomly assigned to one out of two groups (control/experimental). The experimental group will complete a personalized CCT intervention based on CogniFit’s Personalized Online Training, including a series of games and activities targeting attention, memory and executive functions. The difficulty level of each of the activities will dynamically change to match the cognitive profile of each participant on each training day. By automatically adjusting the activities’ difficulty and requirements according to each participant’s performance, cognitive taxing is adapted and a maximum cognitive effort is always required. Similar to Haimov and Shatil (2013), the active control group will complete a series of painting activities specifically designed not to result cognitively demanding and to maintain a low complexity level and constant difficulty throughout the intervention. These activities will not target the intended cognitive processes (namely, they will target orthogonal unrelated aspects). Participants will be randomly assigned to either the experimental or control group, with the experimenters and the patients being fully blind to the group classification.

After post-test data collection, and prior to statistical analyses, all variables will be checked for normality of distribution, and plots will be visually inspected for asymmetry and kurtosis. Then, descriptive statistics will be obtained to provide an overview of the sociodemographic and clinical characteristics of the participants. To examine intervention effectiveness, the changes in all outcome measures from baseline (pretest) to post-intervention (post-test) will be analyzed. Differences in primary outcomes and secondary outcomes will be analyzed through linear mixed models for repeated measures. Models will assess within and between-group differences before and after the intervention. The Group factor (experimental, control) will be included as an independent variable, together with Time (pre-test at baseline, post-test after the intervention). Sociodemographic factors such as gender and age will be used as moderators. Dependent variables will be sleep quality, insomnia severity, quality of life, cognitive abilities, executive function, depressive symptoms, anxiety symptoms, and worrying. To isolate the variable that better explains the model, a hierarchical regression analysis will be conducted with the sleep quality improvements as the dependent variable and the cognitive improvements as the independent ones. This hierarchical regression analysis will be also useful to evaluate the impact of the adherence to the training in the whole sample of participants, regardless of whether they have effectively completed the intervention or not (i.e., cases of attrition or incomplete sessions). The hierarchical regression analysis with the sleep quality improvement as the dependent variable will follow a stepwise approach in which sociodemographic factors will be added in a first step, the data corresponding to the adherence to the treatment (namely, number and length of the completed sessions) in a second step, and the cognitive data from the BRIEF-A and the Cognitive Assessment Battery CAB™ as a third and final step.

### Participant Selection and Sample Sizes

Participants will be recruited from the Sleep Unit of the Hospital Universitario de la Ribera (Spain) for the phase-I and phase-II trials, and from the same Unit and associated medical centers for the phase-III trial. According to the *a priori* established eligibility criteria, participants will be adults between 25 and 55 years old who have been diagnosed by Insomnia Disorder according to DSM-5, fully matching the diagnostic criteria (e.g., showing a predominant complaint of dissatisfaction with sleep quantity or quality occurring at least three nights per week and having been present for a minimum of 3 months, despite them having adequate opportunity for sleep and in the absence of sleep-wake disorders or the use of substances, which causes clinically relevant impairment). We selected this age range in an attempt to improve sample homogeneity in executive function development (see Ferguson et al., 2021), and to ensure that participants would have access to new technologies. Participants will be excluded from the study if they meet any of the following *a priori* established criteria: (1) they present another sleep-wake disorder (e.g., narcolepsy, restless leg syndrome, a breathing-related sleep disorder, a circadian sleep-wake rhythm disorder, a parasomnia); (2) they present a relevant medical, psychiatric or neurological disorder; (3) they show significant visual or motor impairments; (4) they present history of alcohol or drug abuse or dependence; (5) they show a caffeine consumption higher than 150 mg per day or an alcohol consumption higher that 250 ml per day; (6) they use medication with stimulant action, except the sedatives or hypnotics specifically prescribed for sleep.

The phase-I trial will follow a standard 3 + 3 protocol. Consequently, the minimum number of participants will be three, and the maximum number of participants will depend on the results of each of the 3-patient cohorts. The phase-II trial will include 20 insomnia patients. Finally, the phase-III trial will consist of a minimum sample size set at 60 participants per group, given that the purpose of this research is to make a first approach to the positive impact of a cognitive training of executive functions on insomnia and to be able to generalize the results obtained with a 95% confidence interval and sufficient statistical power to test hypotheses (sig. bilateral). While a larger number of participants would be desirable for a phase-III trial, it should be considered that the specific selection criteria of the sample and the restricted number of patients that are available at the hospital and the associated centers make it difficult to increase the sample size to account for potential attrition rates and lack of adherence.

### Instruments and Outcome Measures

We will carry out a specific evaluation of sleep quality and insomnia, together with a general evaluation of the participant’s neurocognitive and emotional state. Likewise, these evaluations will be complemented with an assessment of the quality of life.

#### Primary Outcome Measures

##### Sleep Quality

The Pittsburgh Sleep Quality Index (PSQI) will be used. The PSQI is a self-report questionnaire that assesses sleep quality and consists of 19 individual items, creating seven components that produce one global score. It takes 5–10 min to complete. The PSQI includes a scoring key for calculating a patient’s seven sub-scores, each of which can range from 0 to 3. The sub-scores are tallied, yielding a global score that can range from 0 to 21. A global score of five or more indicates poor sleep quality, and the higher the score is, the worse the sleep quality can be considered.

##### Insomnia Severity

The Insomnia Severity Index (ISI) will be used. The ISI is a brief instrument designed to assess the severity of both nighttime and daytime components of insomnia. It comprises seven items assessing the perceived severity of difficulties initiating sleep, staying asleep, early morning awakenings, satisfaction with current sleep pattern, interference with daily functioning, noticeability of impairment attributed to the sleep problem, and degree of distress or concern caused by the sleep problem. Possible scores range from 0 (no clinically significant insomnia) to 28 (clinical severe insomnia).

##### Quality of Life

The Measuring Quality of Life | The World Health Organization–abridged version (WHOQOL-BREF) will be used. The WHOQOL-BREF is a generic questionnaire to measure the quality of life created by the Study Group on Quality of Life of the World Health Organization. It has two general questions on the quality of life and satisfaction with the state health, and 24 questions grouped into four areas or domains: Physical Health, Psychological Health, Social Relations, and Environment. The measure is calculated by summing the scores in the questions corresponding to each domain and then transforming them to a 0–100 point interval. Possible scores for each domain range from 0 (poor perceived quality of life) to 100 (greater perceived quality of life).

Additionally, the short version of the self-perceived global physical and mental health scale from the Patient-Reported Outcomes Measurement Information System (PROMIS v.1.2; see (Hays et al., 2015, 2017), will be also used. Participants will respond to the following questions: *In general, how would you rate your health?* (Options: Excellent, Very Good, Good, Fair, Poor); *In general, how would you rate your physical health?* (Options: Excellent, Very Good, Good, Fair, Poor); *To what extent are you able to carry out your everyday physical activities such as walking, climbing stairs, carrying groceries, or moving a chair?* (Options: Completely, Mostly, Moderately, A little, Not at all); *In general, how would you rate your mental health, including your mood and your ability to think?* (Options: Excellent, Very Good, Good, Fair, Poor); *In general, how would you rate your satisfaction with your social activities and relationships?* (Options: Excellent, Very Good, Good, Fair, Poor).

#### Secondary Outcome Measures

##### Executive Functions

The Behavior Rating Inventory of Executive Function-Adult Version (BRIEF-A) will be used. The BRIEF-A is a standardized measure that captures views of an adult’s executive functions or self-regulation in his or her everyday environment. Only the self-report format will be used. The BRIEF-A comprises 75 items within nine theoretically and empirically derived clinical scales that measure various aspects of executive functioning; Inhibit, Self-Monitor, Plan/Organize, Shift, Initiate, Task Monitor, Emotional Control, Working Memory, Organization of Materials. The clinical scales form two broader indexes: Behavioral Regulation (BRI) and Metacognition (MI), and these indexes form the overall summary score, the Global Executive Composite (GEC). It also includes three validity scales (Negativity, Inconsistency, and Infrequency). It takes approximately 10–15 min to administer. All 75 items are rated in terms of frequency on a 3-point scale: 0 (never), 1 (sometimes), 2 (often). Raw scores for each scale are summed for the total score.

##### Cognitive Skills

The Cognitive Assessment Battery CAB™ test provides a general cognitive score as well as specific scores in each of the measured cognitive skills. The Cognitive Assessment Battery CAB™ includes a series of 17 short tests (∼2–3 min each) that measure a variety of different cognitive abilities, putting a heavy focus on executive functions. These are then used to obtain a general score, as well as five different sub-scores based on perception, attention, memory, coordination and reasoning. The resulting scores per participant in each of the 23 cognitive skills measured by the CAB™ are contrasted with the normative database of the test and each converted into z-scores and percentiles. It takes about 40 min to administer when done for the first time prior to any form of computerized training, and then it automatically updates the information and scores by recalculating the output as a function of participants’ performance in the tasks included in CogniFit’s Personalized Online Training.

##### Depressive Symptoms

The Beck Depression Inventory-II (BDI-II) will be used. The BDI-II is a 21-item, self-report inventory designed to measure the frequency and severity of depressive symptoms. Items include somatic-affective symptoms as well as cognitive symptoms. Possible scores range from 0 (no depressive symptoms) to 63 (severe depression).

##### Anxiety Symptoms

The State-Trait Anxiety Inventory (STAI) will be used. The STAI is a self-report that assesses two types of anxiety: state anxiety, or anxiety about an event, and trait anxiety, or anxiety level as a personal characteristic. Only the 20 items referred to state anxiety will be administered. Possible scores range from 0 (no anxiety) to 60 (severe anxiety).

##### Worrying

The Penn State Worry Questionnaire (PSWQ) will be used. The PSWQ is a 16-item questionnaire that aims to measure the trait of worry. The items on the scale assess the occurrence, intrusiveness, pervasiveness, and other characterizing features of an individual’s experience with worry. The scale has been shown to identify worry, over and above anxiety and depression. Items are rated on a five-point scale: 1-Not at all typical of me to 5-Very typical of me. Possible scores range from 16 (low worry) to 80 (high worry).

#### Safety Outcome Measures^1^

##### Fatigue

Participants will respond to a first question targeting the fatigue level felt at the time of finishing the session, adapted from the Brief Fatigue Inventory (Mendoza et al., 1999): *Please rate your fatigue (weariness, tiredness) by selecting the number that best describes your fatigue right now* [Options: Scale from 0 (no fatigue) to 10 (as bad as you can imagine)].

##### Adverse Events

The safety questionnaire will include a definition of side effects as any effect that was not the intended clinical effect of the CCT, regardless of it being harmful or adverse. Participants will respond to a binary question (yes/no) to indicate whether they feel any side effect right after the session. Similarly, all participants will be also asked a binary (yes/no) question about possible adverse events they may feel. Adverse events will be defined as any undesirable experience associated with the use of the CCT platform: *Please indicate if you feel any side effect of the Computerized Cognitive Training session you have just finished, regardless of it being harmful or adverse* (Options: Yes, No); *Please indicate if you feel any undesirable experience associated with the Computerized Cognitive Training* (Options: Yes, No).

In case any participant at any moment would respond “yes” to any of the two safety questions, a member of the research team would immediately interview them in order to classify the side effect or adverse event according to its seriousness following the criteria and elements adapted from the Patient-Reported Adverse Drug Event Questionnaire (de Vries et al., 2013).

## Expected Results

We expect results to show between-group significant improvements for the cognitive stimulation training group (namely, the experimental group completing the CCT intervention) on sleep quality, quality of life, and cognitive performance. Moreover, due to the relationship between cognitive performance—mainly of cognitive functions—and cognitive awareness and distraction avoidance, we also anticipate a reduction in depressive and anxiety symptoms, and cognitive worrying, all of these linked to a reduced insomnia severity. The fact of receiving the treatment could cause minor improvement in the active control group. Being immersed in the intervention program and carrying out the proposed activities daily—even if they are not designed for it—could lead to an improvement in cognitive functioning. However, if it were to occur, it would not be expected that this improvement would be significant, or if it were, it would be expected to occur in cognitive functions not related to the pathology under study (i.e., verbal fluency).

## Discussion

This project represents an advance in cognitive and health science research and presents a transfer potential of great relevance. The results hope to offer a viable and accessible intervention that aims to enhance the cognitive performance and the quality of life of those who suffer from sleep disturbances. If the results are as expected, this study may encourage the implementation of home-based personalized cognitive stimulation interventions for patients with insomnia and sleep disturbances. This intervention could decongest the health system, improve patients’ care and quality of life, reduce the side effects of pharmacological treatment, and save on medical care. In order to overcome clinician barriers briefly mentioned above (i.e., lack of knowledge of non-pharmacological interventions by clinicians), planned dissemination of the results would be necessary.

## Data Availability Statement

The original contributions presented in the study are included in the article/supplementary material, further inquiries can be directed to the corresponding authors.

## Ethics Statement

This study was reviewed and approved by the Comité de Ética en Investigación—CEI (UNNE-2021-006) and the Comité de Ética de la Investigación of the Generalitat Valenciana, Consellería de Sanitat Universal i Salut Pública. The patients/participants provided their written informed consent to participate in this study.

## Author Contributions

All authors listed have made a substantial, direct, and intellectual contribution to the work, and approved it for publication.

## Conflict of Interest

The authors declare that the research was conducted in the absence of any commercial or financial relationships that could be construed as a potential conflict of interest.

## Publisher’s Note

All claims expressed in this article are solely those of the authors and do not necessarily represent those of their affiliated organizations, or those of the publisher, the editors and the reviewers. Any product that may be evaluated in this article, or claim that may be made by its manufacturer, is not guaranteed or endorsed by the publisher.

## Footnotes

1 Note that the safety questionnaire exclusively applies to the phase-I and phase-II trials.
